# Global Systems-Level Analysis of Hfq and SmpB Deletion Mutants in *Salmonella*: Implications for Virulence and Global Protein Translation

**DOI:** 10.1371/journal.pone.0004809

**Published:** 2009-03-11

**Authors:** Charles Ansong, Hyunjin Yoon, Steffen Porwollik, Heather Mottaz-Brewer, Brianne O. Petritis, Navdeep Jaitly, Joshua N. Adkins, Michael McClelland, Fred Heffron, Richard D. Smith

**Affiliations:** 1 Biological Sciences Division, Pacific Northwest National Laboratory, Richland, Washington, United States of America; 2 Department of Molecular Microbiology and Immunology, Oregon Health and Sciences University, Portland, Oregon, United States of America; 3 Environmental Molecular Sciences Laboratory, Pacific Northwest National Laboratory, Richland, Washington, United States of America; 4 The Sidney Kimmel Cancer Center, San Diego, California, United States of America; University of Minnesota, United States of America

## Abstract

Using sample-matched transcriptomics and proteomics measurements it is now possible to begin to understand the impact of post-transcriptional regulatory programs in Enterobacteria. In bacteria post-transcriptional regulation is mediated by relatively few identified RNA-binding protein factors including CsrA, Hfq and SmpB. A mutation in any one of these three genes, *csrA*, *hfq*, and *smpB*, in *Salmonella* is attenuated for mouse virulence and unable to survive in macrophages. CsrA has a clearly defined specificity based on binding to a specific mRNA sequence to inhibit translation. However, the proteins regulated by Hfq and SmpB are not as clearly defined. Previous work identified proteins regulated by *hfq* using purification of the RNA-protein complex with direct sequencing of the bound RNAs and found binding to a surprisingly large number of transcripts. In this report we have used global proteomics to directly identify proteins regulated by Hfq or SmpB by comparing protein abundance in the parent and isogenic *hfq* or *smpB* mutant. From these same samples we also prepared RNA for microarray analysis to determine if alteration of protein expression was mediated post-transcriptionally. Samples were analyzed from bacteria grown under four different conditions; two laboratory conditions and two that are thought to mimic the intracellular environment. We show that mutants of *hfq* and *smpB* directly or indirectly modulate at least 20% and 4% of all possible *Salmonella* proteins, respectively, with limited correlation between transcription and protein expression. These proteins represent a broad spectrum of *Salmonella* proteins required for many biological processes including host cell invasion, motility, central metabolism, LPS biosynthesis, two-component regulatory systems, and fatty acid metabolism. Our results represent one of the first global analyses of post-transcriptional regulons in any organism and suggest that regulation at the translational level is widespread and plays an important role in virulence regulation and environmental adaptation for *Salmonella*.

## Introduction

Post-transcriptional regulation of gene expression by small noncoding RNAs (sRNAs) is widespread in both prokaryotes and eukaryotes and has only recently been recognized as playing a prominent role in the regulation of cellular processes. In bacteria, regulatory sRNAs act via base-pairing interactions with their target mRNAs to modulate their translation and/or decay [1], [2], in a reaction typically dependent on the RNA-binding protein Hfq [3], [4]. While the role of DNA-binding proteins, particularly transcription factors, in regulating gene expression at the level of transcription has received much attention, how RNA-binding proteins control gene expression at the post-transcriptional level remains unclear.

The RNA-binding protein Hfq was initially shown to be the host factor required for replication of Qβ RNA bacteriophage [5] and is a member of the Sm protein family of RNA binding proteins that also include eukaryotic proteins required for mRNA splicing [6], [7]. Hfq is a highly conserved protein encoded within many bacterial genomes and at least one archaeon genome [7], [8]. In bacteria, Hfq functions as a post-transcriptional regulator that modulates the stability and translation of mRNAs chiefly by facilitating sRNA-mRNA interactions [1], [4], [9]. Its broad role in regulation at the translational level has recently been hinted at by Sittka and co-workers [10].

The RNA-binding protein SmpB specifically interacts with the sRNA *tmRNA* (also known as *ssrA* and *10Sa*) and is required for all known functions of *tmRNA* [11]. Indeed SmpB deletion mutants have been observed to have the same phenotype as *tmRNA* deletion mutants [11]. *tmRNA* is found in a wide range of bacteria and in the mitochondria of some eukaryotes and uniquely exhibits properties of both tRNA and mRNA [12], [13], [14]. *tmRNA* functions as a part of the translational quality control process, recognizing and binding to ribosomes that become stalled. Once bound, *tmRNA* adds a peptide tag to the nascent partially synthesized protein chain which targets it for degradation by C-terminal-specific cellular proteases [15], [16], [17], [18]. Usually classified as a housekeeping or repair system, a role for *tmRNA* as a regulatory sRNA has been recently suggested. Ranquet and Gottesman [19] report that the sRNA *tmRNA* plays a regulatory role in gene expression and is required for the correct high-level translation of RpoS.

Recent reports suggest that both Hfq and SmpB play essential roles in bacterial pathogenesis. For example an Δ*hfq* mutant derivative in the pathogen *Salmonella enterica* serovars Typhimurium (*Salmonella* Typhimurium) was found to attenuate virulence in mice and was impaired in the invasion of non-phagocytic cells [20]. Hfq was also reported to be essential for *Vibrio cholerae* virulence, with sRNAs in conjunction with Hfq suggested as likely critical regulators of *Vibrio cholerae* pathogenicity [21]. Robertson and Roop demonstrated that a *Brucella abortus* Δ*hfq* mutant failed to replicate in cultured murine macrophages, and displayed attenuated virulence in a mouse model [22]. In another study, a *smpB* mutant of *Salmonella* Typhimurium exhibited defects in intra-macrophage survival, a model of *Salmonella* pathogenesis [23]. More recently mutation of *tmRNA* was shown to be responsible for a marked reduction in *Salmonella* Typhimurium virulence in BALB/c mice [24]. In *Yersinia pseudotuberculosis* mutations of *smpB* and *tmRNA* generated a bacterium that exhibited defects in survival and replication in macrophages and was avirulent in mice [25]. Collectively, these results demonstrate that Hfq and SmpB are essential for regulating virulence by controlling translation of key components.

Identifying what proteins are actually regulated by Hfq and SmpB is an important step in understanding the impact of post-transcriptional gene regulation in Enterobacteria and more specifically how Hfq and SmpB control *Salmonella* gene expression at the post-transcriptional/ translational level and the role that this may play in *Salmonella* pathogenesis. Towards the global identification of Hfq targets, previous efforts have employed a combination of co-immunoprecipiatation (co-IP) and or microarray analysis. Zhang et al. [26] used high-density microarrays to detect potential targets of Hfq co-immunoprecipitated with Hfq-specific antibodies. In a recent study Sittka et al. [10] used high-throughput pyrosequencing (HTPS) to detect potential targets of an epitope tagged-Hfq protein co-immunoprecipitated with commercially available antibodies, overcoming the limitations of the previous strategy; the need for custom microarrays and specialized antibodies. In another study [27] microarray analysis was used to identify changes in gene expression resulting from lack of Hfq, thus providing potential new target genes for sRNA regulation. While co- immunoprecipiatation has proven valuable in identifying targets of Hfq, and thus targets of translational regulation, one possible criticism of this approach is that the RNA co-immunoprecipitated with Hfq may be brought down simply because Hfq strongly binds to RNA molecules and that the binding is somewhat non-specific. In addition, it is clear that a transcriptional analysis solely can not distinguish between transcriptional and translational/post-transcriptional regulatory effects.

Using sample-matched transcriptomics and proteomics measurements it is possible to accurately characterize on a global scale control of gene expression at the post-transcriptional level. Here, we performed sample-matched global proteomics and transcriptional analyses together with cellular assays and animal studies to begin to understand how Hfq and SmpB control *Salmonella* gene expression at the post-transcriptional level and the role that this may play in *Salmonella* pathogenesis. Our global analyses revealed that a relatively high percentage of all the annotated *Salmonella* genes (>20%) are regulated post-transcriptionally either directly or indirectly. The extent of global regulation of translation is much greater than previously thought [10], with profound effects in all stages of *Salmonella's* life cycle including a variety of house keeping pathways and known and novel virulence factors, in part explaining the requirement of both translational regulators for virulence in *Salmonella*. This study represents one of the first comparative global analyses of post-transcriptional regulation in any organism; the data suggest post-transcriptional regulation is widespread and plays an important role in virulence regulation and environmental adaptation for *Salmonella*.

## Results and Discussion

### Construction and growth characteristics of mutant and wildtype *Salmonella* Typhimurium strains

Hfq and SmpB are essential for virulence in a variety of pathogenic bacteria including *Salmonella* Typhimurium [20], [21], [22], [25]. To address the role of Hfq and SmpB in *Salmonella* Typhimurium pathogenesis and protein synthesis we first constructed non-polar deletion mutations of the *hfq* and *smpB* genes using a modified pKD13 derivative as the template. In this method [28], the coding region between the initiation methionine and the last seven amino acids of the gene was replaced with an inframe “scar” sequence that does not contain any stop codons. Functional disruption of the *hfq* and *smpB* mutant derivatives was first confirmed by PCR analysis after which the mutants were P22 transduced to a new genetic background to avoid the possibility of secondary mutations. Mutants that lacked either of these proteins showed impaired growth phenotype in acidic minimal media (AMM). The *hfq*-deficient strain also exhibited decreased growth rate in Luria-Bertani (LB) broth (Figure S1). These results suggest SmpB and Hfq are involved in broad cellular functions, including growth rate regulation.

### Δhfq and ΔsmpB *Salmonella* Typhimurium mutant strains are avirulent

**Figure 1:**
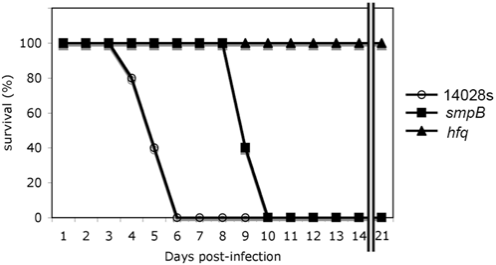
Comparison of survival in mice. Five BALB/c mice were i.p. infected with 200 cfu of *Salmonella* for each strain; the wild type strain, the Δ*hfq* mutant strain and the Δ*smpB* mutant strain, and monitored for 21 days. Alive mice were counted and shown in % survival.

To directly assess the role of Hfq and SmpB in *Salmonella* Typhimurium pathogenesis, BALB/c mice were infected intraperitoneally with 200 cfu of either Δ*hfq* mutant strain, Δ*smpB* mutant strain or wildtype strain and monitored for 21 days (Figure 1). The absence of *smpB* and *hfq* attenuated *Salmonella* virulence; mice infected with the Δ*smpB* mutant strain died at 10 days post-infection while mice infected with the Δ*hfq* mutant strain were fully resistant to the infection. Mice infected with wildtype strain exhibited typical symptoms of infection and died at around six days. These results are similar to those in previous reports [20], [23].

### Δhfq and ΔsmpB *Salmonella* Typhimurium mutant strains show defects in intra-macrophage proliferation

**Figure 2:**
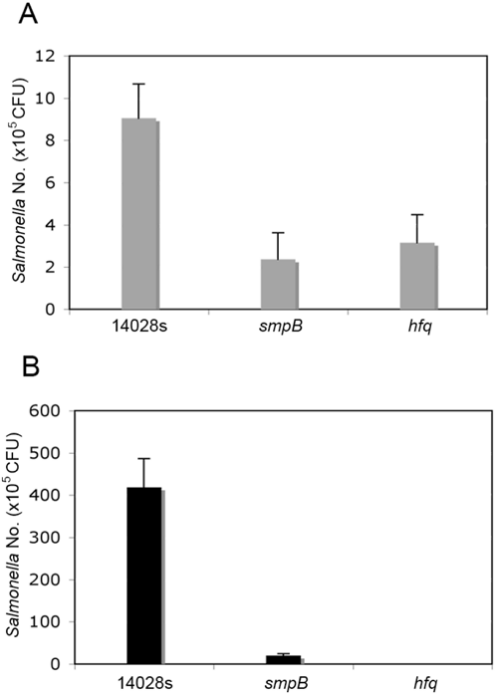
Comparison of survival within macrophage cells. RAW264.7 cells were infected with *Salmonella* cells grown overnight in LB via MOI of 50. After infection initiation by centrifuging the bacteria onto the cell monolayers at 1,000×g for 5 min, cells were incubated at 37°C with 5% CO_2_ for 30 min, treated with gentamicin (100 µg/ml) for 1 h, and incubated in DMEM with gentamicin (20 µg/ml) for the remainder of the test. Macrophage cells were lysed using 1% Triton-X at 1.5 h (A) and 18 h (B) post-infection and the intracellular bacteria were enumerated by serial dilution of cell lysates. The data shown represents the mean and standard deviation of three individual experiments.

As *Salmonella*'s ability to replicate within macrophages is essential for its virulence [29], we examined intracellular replication of Δ*hfq* and Δ*smpB Salmonella* Typhimurium mutant strains within the macrophage- like cell line RAW264.7. These cells were infected at a multiplicity of infection (m.o.i.) of about 1 bacteria per cell with stationary phase bacteria grown overnight in LB broth as described in Materials and Methods. Extracellular bacteria were removed by washing and treatment with gentamicin. After infection the cells were lysed at various times by rapid resuspension in PBS containing 1% Triton X-100. Intracellular survival/replication results (Figure 2) are consistent with earlier reports that suggest Δ*hfq* and Δ*smpB Salmonella* Typhimurium mutant strains are essential for intra-macrophage proliferation [22], [23], [25].

### Global effects of Hfq and SmpB mutation on *Salmonella* Typhimurium chromosome

LC-MS-based proteomics was used to obtain a general overview of the effects of Hfq and SmpB on global protein translation in *Salmonella*. The differences in protein expression between parent and isogenic mutant was assessed via hypothesis testing, specifically a comprehensive ANOVA scheme included in the computer program DAnTE [30]. Relative changes in protein abundance between Δ*smpB* or Δ*hfq* mutant strains and a wild-type strain were evaluated across four differing growth conditions. These included logarithmic (log) and stationary (stat) phase cultures and growth in two variations of an acidic, low magnesium [Mg^2+^] minimal media (AMM), to approximate conditions encountered in the hosts's phagocytes (Materials and Methods). Across all experimental datasets we observed a total of 1621 *Salmonella* Typhimurium proteins (Table S1), corresponding to ∼36% coverage of predicted protein coding regions. These proteins represent a broad spectrum of gene products from the genetic translation (Table 1), with proteins of unknown or unclassified function representing the largest functional category as might be expected.

**Table 1:** JCVI functional categories for the entire annotated genome and characterized proteome.

Functional Categories	Total from genome	Percent of genome	Total of observed proteome	Percent of observed proteome	Total of observed proteome	Hfq regulated	SmpB regulated	% Hfq regulated	% SmpB regulated
Amino acid biosynthesis	130	2.69	84	4.86	84	37	15	44.05	17.86
Biosynthesis of cofactors, prosthetic groups, and carriers	163	3.37	93	5.38	93	34	6	36.56	6.45
Cell envelope	469	9.71	131	7.58	131	58	10	44.27	7.63
Cellular processes	286	5.92	117	6.77	117	74	22	63.25	18.80
Central intermediary metabolism	168	3.48	86	4.97	86	42	17	48.84	19.77
DNA metabolism	155	3.21	56	3.24	56	27	7	48.21	12.50
Energy metabolism	546	11.30	250	14.46	250	149	24	59.60	9.60
Fatty acid and phospholipid metabolism	73	1.51	36	2.08	36	20	1	55.56	2.78
Hypothetical proteins	78	1.61	27	1.56	27	9	1	33.33	3.70
Mobile and extrachromosomal element functions	221	4.58	17	0.98	17	8	6	47.06	35.29
Protein fate	184	3.81	93	5.38	93	45	14	48.39	15.05
Protein synthesis	375	7.76	146	8.44	146	67	15	45.89	10.27
Purines, pyrimidines, nucleosides, and nucleotides	81	1.68	64	3.70	64	33	5	51.56	7.81
Regulatory functions	304	6.29	82	4.74	82	34	5	41.46	6.10
Signal transduction	26	0.54	7	0.40	7	2	0	28.57	0.00
Transcription	52	1.08	31	1.79	31	13	4	41.94	12.90
Transport and binding proteins	517	10.70	113	6.54	113	61	15	53.98	13.27
Unclassified	332	6.87	94	5.44	94	50	11	53.19	11.70
Unknown function	670	13.87	202	11.68	202	99	27	49.01	13.37

Genes and gene products are categorized by the JCVI (J. Craig Venter Institute), formerly TIGR (The Institute for Genomic Research), categorization system. Some proteins are annotated to multiple categories, accounting for the larger total protein count.

Across each of the four growth conditions examined our analysis revealed that Hfq had a far more significant effect on the *Salmonella* Typhimurium proteome than SmpB. Overall, in comparison to expression in the parent strain, Hfq and SmpB altered, ∼50% (781proteins) and ∼12% (189 proteins) of the observed proteome (1621 proteins) respectively, as determined by an ANOVA analysis (p<0.05) using the computer program DAnTE [30] (Table S1). When compared to the entire *Salmonella* proteome of 4450 annotated open reading frames (orf; [31]), 781 proteins represents ∼20% of the entire *Salmonella* genome. This is in agreement with a recent study that found that Hfq affects 18% of the *Salmonella* genome [10]. Considering the reported pleitropic nature of deletion of *hfq* on the biology of *Salmonella* Typhimurium it is likely that a proportion of the changes in protein abundances maybe a non-specific and/or secondary consequence of *hfq* deletion. That is, not every deregulated protein in the *hfq* mutant strain is necessarily directly regulated by Hfq. Indeed analysis of the correlation between Hfq-dependent proteins identified in this study and Hfq-associated mRNAs identified by Sittka et al. [10] showed an approximately 25% overlap (Table S2), similar to the ∼30% overlap observed between data from a transcriptional analysis of an Δ*hfq* mutant strain and data from a co-IP analysis of Hfq-associated mRNAs [10].

We report that Hfq directly or indirectly modulates ∼20% of the *Salmonella* genome. However this most likely is an underestimate of the true number of proteins that are regulated by Hfq, if accurate protein identifications could be made for the remaining 2829 orfs of the entire *Salmonella* proteome (4450 orfs) that are either expressed at levels too low to be detected or not expressed under the four growth conditions employed in this study. Considering that we observe 36% (1621 orfs) of the entire *Salmonella* proteome (4450 orfs) and that 50% (781 orfs) of this observed proteome (1621 orfs) is modulated by Hfq, it would be expected that a significant portion of the unobserved *Salmonella* proteome (2829 orfs) would also be regulated by Hfq, thus our assertion that the ∼20% value may very well represent an underestimation. Similarly 189 proteins represents ∼4% when compared to all annotated orfs but again this is likely an underestimate of proteins regulated by SmpB if accurate protein identifications could be made for the remaining 2829 orfs that are either expressed at levels too low to be detected or not expressed under the four growth conditions employed in this study.

Classification of the *Salmonella* proteins deregulated by either Hfq or SmpB by the J. Craig Venter Institute (JCVI) functional categories [32] showed that Hfq modulated the expression of ≥40% of the observed proteins in 15 of the 19 functional categories annotated for *Salmonella* (Table 1). The “Cellular Processes” functional category represented the category with most genes impacted by Hfq (∼63%). This category encompasses sub-categories including adaptations to atypical conditions, cell adhesion, pathogenesis, chemotaxis and motility, detoxification, toxin production and resistance, DNA transformation and cell division. Translational regulation of these genes permits more rapid adaptation than would be required if the genes had to be first transcribed. This observation may explain the acute attenuation in virulence observed for the Δ*hfq* mutant as well as the previously observed invasion and motility phenotypes of an Δ*hfq* mutant [10], [20].

We validated a representative set of proteins by comparing our results with published results. Sittka et al. [20] investigated the proteome of an Δ*hfq* mutant strain using 2D gel analysis, and found ∼70 differentially expressed proteins in early stationary phase. Across all growth conditions we identified 67 of the ∼70 differentially expressed proteins identified in that study. Furthermore 51 of the ∼70 Hfq-dependent proteins identified by Sittka et al. were also found to be differentially expressed in the present study. The direction of regulation of many of these proteins was in general agreement with the literature including OmpF, DppA, FliC, SurA, and LuxS as shown in Table S3. As best as we can approximate our LB Stat growth condition would most closely match the early stationary phase growth condition described by Sittka et al. [20] and hence one would expect minimal variability in a cross-comparison between both studies under these two growth conditions. Indeed the direction of regulation of 21 out of 22 differentially expressed proteins in LB Stat growth is in agreement with the literature providing validation. The exception was the SPI-1 protein SipA under LB Stat growth conditions. However under SPI-1 inducing conditions (LB Log) regulation of SPI-1 proteins by Hfq was generally consistent with the literature. To extend our validation of the proteomics results we also directly analyzed protein levels by Western blot analysis of selected proteins based on our proteomics results. Our proteomics results showed that HtrA protein levels were strongly up-regulated in the Δ*hfq* mutant relative to wildtype in LB Log, while OsmY and STM1513 were strongly down-regulated in the Δ*hfq* mutant relative to wildtype in AMM-1 and LB Stat respectively. Deletion of SmpB resulted in a significant decrease in protein expression levels of FliC under LB Log and a significant increase in the protein levels of YciF in AMM-2. Western blot analysis of the relative protein levels of each of HtrA, OsmY, STM1513, FliC, and YciF (Figure S2) served to further verify our global proteomics measurements.

**Figure 3:**
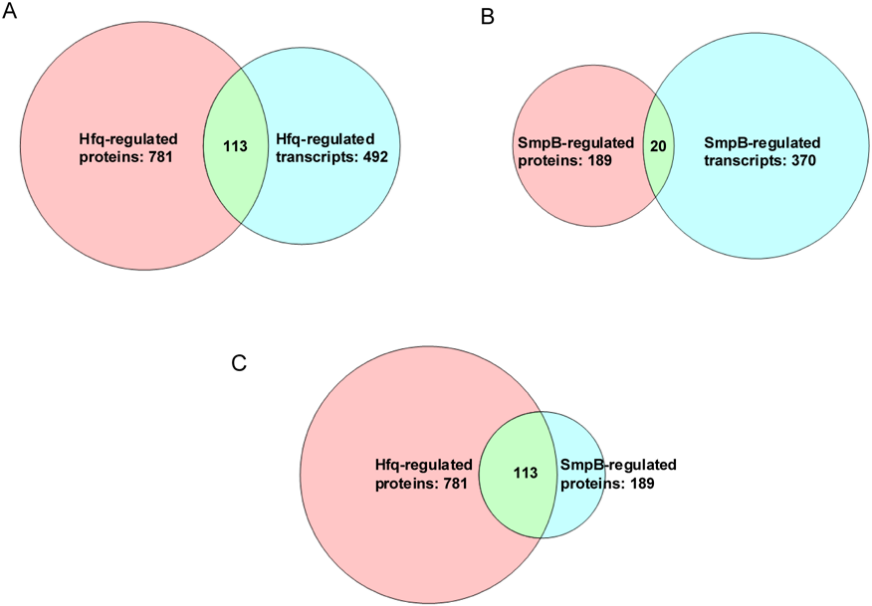
Summary of transcriptional and translational control functions of Hfq and SmpB. Venn diagram showing overlap between transcripts and proteins regulated by Hfq (Panel A), between transcripts and proteins regulated by SmpB (Panel B) and between proteins co-regulated by Hfq and SmpB (Panel C) across all growth conditions.

As transcript levels are often equated to protein expression levels, it was of particular interest to determine whether genes that showed significant changes in protein expression also exhibited significant changes in transcript levels. Examination of transcript levels using microarray analysis (≥2-fold change) revealed a more modest effect on *Salmonella* transcription, with ∼11% (492 genes) and ∼8% (370 genes) of the *Salmonella* Typhimurium genome (4550 genes; [31]) under control of Hfq and SmpB, respectively (Table S4). The result for *hfq* transcriptional analysis is in general agreement with previous studies for *Salmonella* [10] and *Pseudomonas aeruginosa* [33] where ∼20% and ∼15% of all genes, respectively, were regulated by Hfq, but slightly higher than the number of Hfq-dependent genes reported for *E. coli* [27] and *Vibrio cholerae* [21], ∼6% each. In light of the fact that these studies employed varying growth conditions it is not possible to perform a rigorous direct comparison across these diverse datasets. However it is worth noting that our observation that Hfq strongly regulates flagellar genes and SPI-1 TTSS genes, discussed below, is consistent with previous results in *Salmonella* [10], [20]. Overall, the transcription data showed fewer discernable patterns of regulation by Hfq or SmpB relative to the proteome analysis, and comparison across all growth conditions revealed little overlap in proteins and transcripts regulated by either Hfq or SmpB (Figure 3). Of the 781 proteins and 492 transcripts regulated by Hfq, 113 genes overlapped both sets. For SmpB-regulated proteins (189) and transcripts (370) there was an overlap of 20 genes. Given the fact that the present study specifically examines the effects of two translational regulators, which decouple transcription and translation, a lack of correlation between transcriptomics and proteomics might be expected. Overall Hfq has a much stronger global effect on *Salmonella* protein translation than SmpB which is in agreement with the large number of regulatory sRNAs that serve as guides for Hfq translational modulation as opposed to the single regulatory sRNA (i.e. *tmRNA*) that requires SmpB for activity.

### Hfq control of biological processes

While the role of Hfq as a global regulator has become increasingly appreciated over the past few years, the actual extent of Hfq regulation of translation remains unknown. We observed changes in expression for ∼50% of the observed *Salmonella* Typhimurium proteome based on an ANOVA analysis (p<0.05) (Table S1). The proteins affected represent a broad spectrum of *Salmonella* proteins required for many biological processes including general metabolism, the translational machinery, stress response, and virulence-assocaited processes as described next.

#### General metabolism

Proteome profiling data suggested Hfq regulates nucleoside (DeoABC) and nucleotide metabolism (CarAB, PurUBCMHAD, Ndk) under AMM conditions, as well as regulates ribose metabolism and transport (RbsABK) under AMM-2. Hfq also appears to play a significant role in central metabolism based on observed differential regulation of numerous proteins involved in glycolysis/gluconeogenesis, the TCA pathway, pyruvate decarboxylation and the pentose phosphate shunt (see Table S1). Other metabolic processes regulated by Hfq include Fatty acid metabolism (FadA, FadB, FabA, FabZ, fbab, AccB, Acs, FabG, FabF, FabI, FabB); Arginine catabolism (AstABCD); and Propanoite catabolism (PrpBCDE). Examination of the transcript levels of the corresponding genes showed no significant change in mRNA expression levels relative to wildtype, suggesting that Hfq provides most of the control of these pathways for the growth conditions monitored.

#### Translation

Hfq also appears to play a role in the regulation of the translational machinery itself presumably as a feedback mechanism to maintain tight control over the amount of ribosomal synthesis. Several ribosomal proteins including RpsM, RpsG, RpmD, and RplN exhibited a marked reduction in both protein and mRNA levels in the Δ*hfq* mutant strain compared to the wildtype under LB Stat growth condition. One hallmark of growth under nutrient poor conditions is lower expression of the machinery necessary to synthesize proteins, including tRNA synthetases and other factors. In fact all of the aminoacyl-tRNA synthetases (IleS, ProS, CysS, GlnS, AspS, ArgS, GltX, AlaS, TrpS, GlyS, GlyQ) observed were up-regulated in the Δ*hfq* mutant relative to the wild type without a concurrent change in transcription. This suggests that Hfq normally represses translation of these genes under conditions that mimics the intracellular environment (AMM).

#### Stress response

Hfq was observed to modulate numerous proteins involved in envelope stress under AMM-2 conditions, including FkpA, SurA, HtrA, NlpB, NmpC, ClpA, SlyD, RseA, RseB, RpoE, OmpF, and HtpG, which were all up-regulated in the Δ*hfq* mutant strain. These observations are in agreement with the recently suggested role of Hfq in envelope stress based on lacZ translational fusions [34] as well as the observation that *rpoE* is translationally regulated by Hfq [10]. Proteins involved in oxidative stress (NarY, Qor, Dps, WraB, OxyR, GrxA, Gor, GrxB, KatG, KatE, SodC, SodB, AhpF); acid stress (CadA); and starvation (SspA, SspB) were all also differentially regulated by Hfq (Table S1). Collectively, these results point to a central role for Hfq in regulating stress response in *Salmonella* Typhimurium. Many of these genes are required for virulence as discussed in more detail below. For example *rpoE* has been shown to be required for the virulence of *Salmonella* Typhimurium [35]. Two RpoE-regulated genes, *htrA* and *surA*, have also been shown to be involved in *Salmonella* virulence [35], [36], [37]. The OxyR-regulated gene *dps* was demonstrated to contribute to *Salmonella* oxidative stress resistance, survival in macrophages, and virulence in mice [38]. Recent studies have shown the periplasmic Cu, Zn-superoxide dismutase *sodC* to contribute to *Salmonella* virulence in mice [39], [40].

#### Virulence-associated

LPS (lipopolysaccharide) has long been known as an essential component of virulence of Gram negative pathogens and, more recently, the agonist for toll like receptor 4. Hfq differentially regulated the expression of several proteins involved in lipopolysaccharides (LPS) biosynthesis in acidic minimal media. LpxA and LpxD involved in Lipid A synthesis, TolC involved in LPS Core completion, as well as proteins involved in O-antigen synthesis (RfbACKMGJU), appeared as up-regulated in the Δ*hfq* mutant strain suggesting that Hfq may normally down regulate expression within the host. Complementary transcriptional analysis revealed no change in the corresponding mRNA levels relative to the wildtype. Reduced expression of LPS and alterations in LPS modification has been reported for intracellular *Salmonella* [41], [42], [43].

Our results suggest Hfq plays a role in the post-transcriptional regulation of several two component systems including three that have been shown to be essential for virulence in a variety of animal models; SsrA/B, PhoP/Q, and OmpR/EnvZ. Two component systems are widespread methods of sensing external conditions and responding by altering gene expression in prokaryotes, eukaryotes and archaea. Well known two component regulators respond to osmolarity, nitrogen and oxygen limitation [44], [45]. As such, they are critical to the ability of pathogenic bacteria to adapt and survive following environmental changes. In addition to the three mentioned above Hfq negatively modulates PhoB involved in the phosphate starvation response, RstA, NarP, RcsB, BasR, and CpxR. Microarray analysis showed no effect on transcription of these genes.

*Salmonella* motility enhances cell contact thereby increasing host cell invasion. A recent study showed that an Δ*hfq* mutant that was non-motile exhibited an invasion defect [20]. Analysis of protein expression in the Δ*hfq* strain showed reduced expression of SPI-1 TTSS components and the flagellar subunit protein FliC compared to the wildtype *Salmonella* strain. Consistent with this previous study, we observed reduced expression of SPI-1 invasion proteins and numerous motility proteins in the Δ*hfq* mutant strain primarily in the log phase growth condition compared to the wildtype strain. Among the SPI-1 invasion proteins were PrgI, SptP, SipD, SicA, InvC and InvG, and the motility proteins FliC, FliT, FliB, CheZ, CheY, and CheW. Microarray results revealed a reduction in mRNA expression of the SPI-1 TTSS and several flagellar proteins, including FliC, FliO, and FliN grown under LB Log growth conditions. This reduction in mRNA expression suggests that Hfq regulates motility and invasion at the transcription level, presumably by modulating one of the upstream regulators. Regulatory elements are generally not made at high levels and thus difficult to identify and quantify. Analysis of our data however demonstrated the upstream regulator for motility *flgM* [46] was poorly expressed in an Δ*hfq* mutant.

#### Propanediol utilization operon

**Figure 4:**
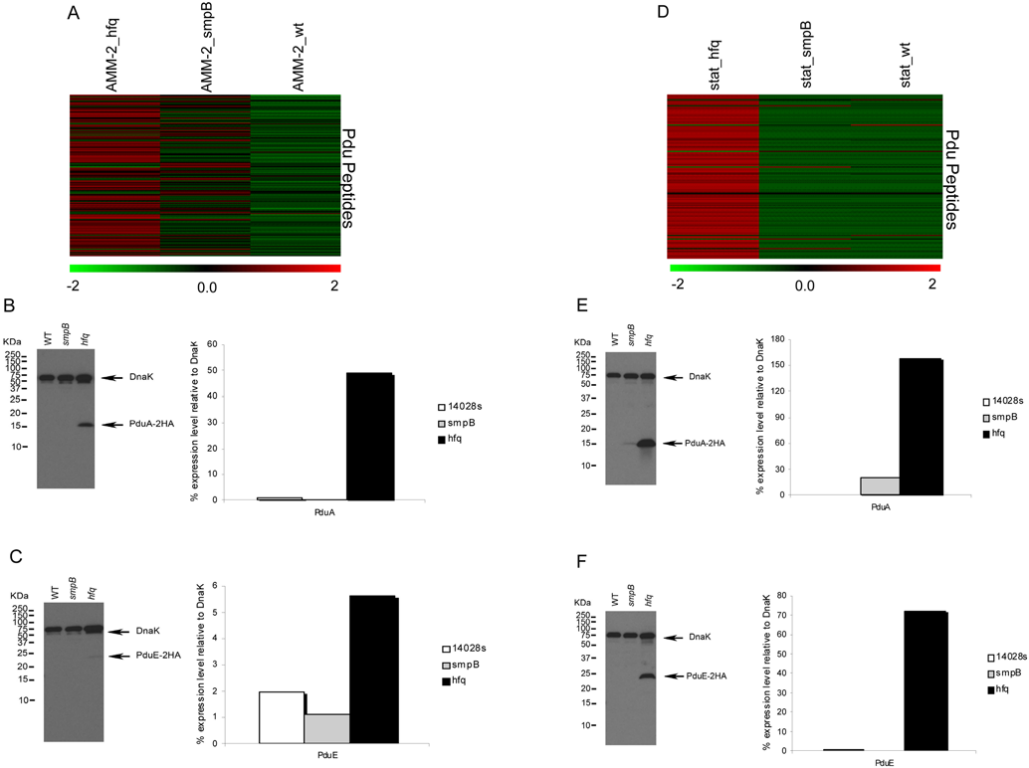
Measurement of protein levels of *pdu* genes in Δ*smpB* and Δ*hfq* strains grown under LB Stat and acidic minimal media conditions. Heatmap showing 134 Pdu peptides that represent 11 individual Pdu proteins up-regulated under acidic minimal media (AMM-2) growth condition (Panel A) and 124 Pdu peptides that represent 11 individual Pdu proteins up-regulated under LB Stat growth condition (Panel D) in Δ*hfq* and Δ*smpB* strains relative to wild-type. *Salmonella* strains harboring HA-tag at each Pdu gene were grown in acidic minimal media (AMM-2) (Panel B and C) and under LB stat phase (Panel E and F) as described. Same amount of cell lysates was loaded in each lane and probed by Western blot analysis for the Pdu-encoded proteins and a control protein DnaK. The level of Pdu-encoded protein expressed in each strain was normalized to the DnaK level. Intracellular DnaK level in each lane was set to 100%.

The *cob* operon is required for the de novo biosynthesis of vitamin B12 in *Salmonella* Typhimurium. Propanediol, derived from rhamnose and fucose catabolism, is degraded by products of the propanediol utilization (*pdu*) operon (immediately adjacent to the *cob* operon) in a vitamin B12 dependent manner. This allows *Salmonella* Typhimurium to grow using propanediol as a sole carbon source and because it is required for growth in macrophages this may be the preferred intracellular metabolite [47]. Results from our proteomics analysis revealed that Hfq regulates the *cob-pdu* operon in AMM-2 (Figure 4A) and stationary phase (Figure 4D) growth conditions, with several products of the *cob-pdu* operon exhibiting increased expression compared to wild type. Among these were PduA, PduB, PduG, PduL, PduM and PduT as well as CobT, CbiH, CbiF, CbiC, and CbiL. This was not apparent in the Δ*smpB* mutant strain in which *cob-pdu* protein levels are the same as the parent (Figure 4A and 4D). Western blot analysis of PduA and PduE relative protein levels validated results from the proteomics analysis (Figures 4B, 4C and 4E, 4F). The strong regulation of Pdu protein expression by Hfq suggests the *pdu* operon to be a potential target of sRNA regulation and that correct coordinate expression may be essential for activity.

**Figure 5:**
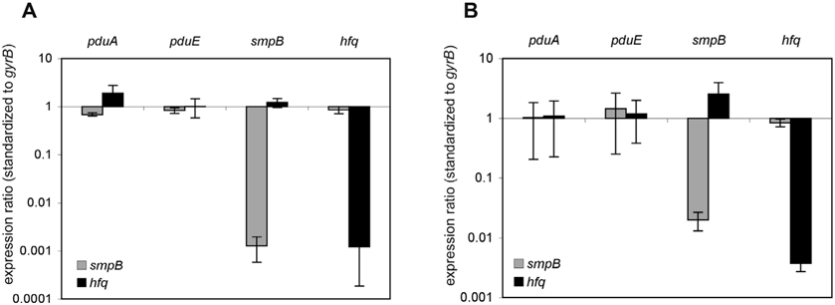
Transcripts of *pdu* genes remain unchanged in Δ*smpB* and Δ*hfq* strains. Total RNA was isolated from *Salmonella* cells grown under acidic minimal media (AMM-2) growth condition (Panel A) or LB Stat growth condition (Panel B) and mRNA level of *pdu* genes was quantified via RT-PCR using *gyrB* mRNA level for normalization. As controls the mRNA level of *smpB* and *hfq* genes were also quantified in a similar fashion. Expression ratios (*smpB* or *hfq*/14028s) were averaged from three individual experiments and shown on a logarithmic scale.

Both microarray analysis and confirmative qRT-PCR assays showed that RNA levels were unchanged for *pduA* and *pduE* genes in the Δ*hfq* mutant strain compared to the wildtype strain (Figure 5), thus showing that Hfq represses translation in these two environmental conditions. The data presented here suggests Hfq likely plays a substantial role in the regulation of the protein expression of the *cob-pdu* operon, required for intra-macrophage replication [47], via post-transcriptional regulatory mechanisms.

### SmpB control of biological processes

SmpB is thought to be limited to trans-translation but has been shown to increase the translation of a single protein - the stationary phase sigma factor RpoS [19]. The normal function of SmpB is in quality control to free ribosomes stalled on defective mRNAs [11], [16]. However, results from our global proteomics analysis indicate that SmpB directly or indirectly modulates the expression of at least 4% of the entire *Salmonella* Typhimurium proteome determined from ANOVA analysis (p<0.05) (Table S1). The majority of genes encoding these proteins showed no changes in mRNA level thus it is interesting to speculate an additional role for SmpB as a general post-transcriptional/translational regulator in *Salmonella* Typhimurium. Few of the genes identified are regulated by RpoS again suggesting that this is a direct translational effect and not indirect transcriptional. The RpoS regulon in *Salmonella* Typhimurium has been initially characterized, identifying 13 conserved RpoS-regulated genes [48]. From our global proteomics analysis we were able to observe gene products for 8 of the 13 conserved RpoS-regulated genes, of which only a single protein, YciF, was regulated by SmpB. We note however that a more detailed and rigorous analysis is required to confirm the speculated global regulatory role of SmpB.

Examination of proteins modulated by SmpB revealed a number of SmpB regulated cellular processes. SmpB regulates histidine biosynthesis in AMM-1, affecting HisDGCBHAF. Proteins involved in motility in *Salmonella* Typhimurium also showed down-regulation during growth in LB media. This set of proteins included the chemotaxis proteins CheZ, CheY, CheW, CheA and Tsr as well as the flagellar proteins FliC, FliD, FliG and FljB. This suggests that a more thorough examination of the role of SmpB in the regulation of cell motility and histidine biosynthesis is warranted. Western blot analysis of FliC relative protein levels validated results from the proteomics analysis (Figure S2B).

### Proteins co-regulated by Hfq and SmpB

We were interested in determining whether there was overlap in translational regulation between Hfq and SmpB i.e., the extent of cross-talk or level of co-ordination if any between these two translational regulators (Table S1). For example it was possible that Hfq directly modulates translation of *smpB* and therefore all of the effects observed for *smpB* could be related to Hfq. For logarithmic phase growth conditions, the expression pattern of 242 proteins appeared to exhibit a dependence on Hfq, while 37 proteins exhibited a dependency on SmpB. Twenty proteins overlapped both sets, including the motility proteins FliC, CheA, CheZ and Tsr. Under stationary phase growth conditions 270 proteins appeared to be under control of Hfq and 27 proteins under control of SmpB. Nine proteins, including the NADPH-dependent quinine oxidoreductase Qor, TolC involved in LPS biosynthesis, and a putative hydrolase YbeM, overlapped. Of the 390 proteins regulated by Hfq and 50 proteins regulated by SmpB in growth in AMM-2, 15 proteins were co-regulated by both Hfq and SmpB, including the invasion protein PrgI, MdoH involved in the synthesis of membrane-derived oligosaccharide, and a putative periplasmic protein CreA. Under growth in AMM-1 267 proteins appeared regulated by Hfq and the expression pattern of 86 proteins exhibited a dependence on SmpB. Twenty four of these proteins were co-regulated by both Hfq and SmpB. This subset included a putative serine/threonine protein kinase, a putative intracellular proteinase YhbO, SrfJ, a known *Salmonella* Typhimurium virulence protein, and the stress protein HtpG. These results also confirm that there is no direct translational control of *smpB* by Hfq.

### Summary

Post-transcriptional regulation has not been directly examined on a global scale by proteomic methods. This work has focused on two translational regulators in *Salmonella* (Hfq and SmpB) that are required for virulence. In order to accurately characterize global control of gene expression at the post-transcriptional level it is essential to measure global changes in both transcript and protein levels. We performed a sample-matched global proteomics and transcriptional analysis to examine the broad impacts of deletion mutants of *hfq* and *smpB* on global protein and transcript abundance levels to begin to understand how Hfq and SmpB mediate the control of global gene expression at the post-transcriptional level. Using a sample-matched procedure our strategy simultaneously measures changes in global transcript and protein abundance levels allowing for a clear distinction between the transcriptional and post-transcriptional effects of Hfq or SmpB. Furthermore, our strategy requires no tagging and or isolation of Hfq or SmpB precluding the possibility of spurious results introduced by tagging and or co- immunoprecipitation processes.

Previous attempts to identify processes/targets regulated by Hfq, and thus targets of sRNA regulation, have relied on transcript-centric approaches. In one study microarray analysis was used to identify changes in gene expression resulting from lack of Hfq, thus providing potential new target genes for sRNA regulation [27]. High-density oligonucleotide microarrays were used to detect sRNA and other targets of Hfq co-immunoprecipitated with Hfq-specific antibodies [26]. Most recently high-throughput pyrosequencing (HTPS) was used to detect message and sRNA bound to Hfq following immunoprecipitation [10]. The result of this work was very surprising namely that Hfq bound to 20% of all *Salmonella* mRNA transcripts and at least 64 small RNA molecules. One possible criticism of this work is that the RNA co-immunoprecipitated with Hfq may be brought down simply because Hfq strongly binds to RNA molecules and that the binding is somewhat non-specific. Our efforts presented here support the conclusions of that study and suggest that an even larger number of *Salmonella* proteins are translationally regulated than the 20% suggested by these authors, when accessed across a larger number of growth conditions. We have compared wildtype and isogenic *hfq* and *smpB* derivatives with matched samples to examine both transcription and translation by global proteomics. There were some cases in which a change in protein level could be related to a change in transcription but this was the exception. These results suggest that while feedback mechanisms between protein abundance and transcript levels exist, the majority of the effects observed here for both *hfq* and *smpB* are a result of direct modulation of post-transcriptional events. These results have been validated by Western blotting to a number of proteins.

The amount of translational regulation varied with growth conditions. During growth in rich media approximately 14% of all proteins observed showed differences in abundance where as in acidic minimal media the result was ∼21%. Taking into account all growth conditions about ∼50% of all observed proteins are regulated by Hfq an astonishingly high number. These results may explain the very high LD50 of the *hfq* mutant strain (>10^9^ i.p.) and its growth defect even in rich media. It has recently been shown that half of all the known sRNAs in *Salmonella* associate with Hfq [10]. It is interesting to note that in adaptive evolution experiments using a chemostat to continuously alter grow conditions often results in changes in Hfq (Bernard Palsson, UCSD, personal communication). It has long been known that many proteins are transcriptionally regulated. These results and the results recently described by Sittka et al. [10] demonstrate that post-transcriptional/translational regulation plays as large a role in controlling the myriad of bacterial processes as does transcriptional regulation. It is the precisely this ability to rapidly adjust to changing conditions in less time than it takes to make an RNA molecule that makes this a pivotal regulatory process.

## Materials and Methods

### Reagents

The following reagents were used in sample preparation: Nanopure or Milli-Q quality water (∼18 megohm·cm or better); ammonium bicarbonate (NH_4_HCO_3_); bicinchoninic acid (BCA) or coomassie protein assay reagents (Pierce, Rockford, IL); urea; thiourea; dithiothreitol (DTT); 3-((3-cholamidopropyl)dimethylammonio)-1-propanesulfonate (CHAPS); calcium chloride; sequencing-grade modified trypsin (Promega); HPLC-grade methanol (MeOH); trifluoroacetic acid (TFA); acetonitrile (ACN); ammonium formate; formic acid; and ammonium hydroxide (NH_4_OH). All reagents were obtained from Sigma Aldrich (St. Louis, MO) unless otherwise specified.

### Bacterial strains and culture conditions

Bacterial strains and plasmids used in this study are listed in Table 2. *Salmonella enterica* serovars Typhimurium strain 14028 wildtype and mutant strains were grown and harvested using standard batch culture procedures. Briefly, cells from a single colony were inoculated into 5 mL of Luria–Bertani (LB) medium and then grown for 16 h with shaking at 37°C. This starter culture was then diluted 1∶100 into 300 mL of fresh LB medium in a 2 L Erlenmeyer flask and grown for 16 h with shaking (200 rpm) at 37°C. Once the logarithmic and stationary phase cultures reached an OD 600 of 0.6 and 2.0, respectively, the cells were harvested via centrifugation at 4000×*g*. The acidic minimal media 1 (AMM-1) cultures used cells pre-cultured in magnesium minimal medium (MgM) pH5.0 overnight at inoculation. Bacteria under stationary phase in MgM media were diluted 1∶100 into fresh pH5.0 MgM and then incubated at 37°C with shaking (200 rpm) for 4 h. For the acidic minimal media 2 (AMM-2) culture, cells were first grown in LB medium to stationary phase as described above, rinsed twice with magnesium minimal medium (MgM) pH 5.0, resuspended in an equal volume of MgM pH 5.0, and then incubated at 37°C with shaking (200 rpm) for 4 h. The cells were harvested as described above.

**Table 2:** Strains and plasmids used in this study.

Strain or plasmid	Description	Ref. or source
*S. enterica* serovar Typhimurium		
14028s	Wild-type	M.J. Worley
HY2688	*smpB*	This study
HY4261	*Hfq*	This study
HYW-pduE	*pduE*-2HA	This study
HYW-pduA	*pduA*-2HA	This study
HYW-htrA	*htrA*-2HA	This study
HYW-STM1513	*STM1513*-2HA	This study
HYW-osmY	*osmY*-2HA	This study
HYW-fliC	*fliC*-2HA	This study
HYW-yciF	*yciF*-2HA	This study
HYS-pduE	*smpB pduE*-2HA	This study
HYS-pduA	*smpB pduA*-2HA	This study
HYS-fliC	*smpB fliC*-2HA	This study
HYS-yciF	*smpB yciF*-2HA	This study
HYH-pduE	*hfq pduE*-2HA	This study
HYH-pduA	*hfq pduA*-2HA	This study
HYH-htrA	*hfq htrA*-2HA	This study
HYH-STM1513	*hfq STM1513*-2HA	This study
HYH-osmY	*hfq osmY*-2HA	This study
Plasmid		
pKD13	rep_R6Kγ_ Ap^R^ FRT Km^R^ FRT	K.A.Datsenko
pKD13-2HA	pKD13 derivative harboring 2HA	This study
pKD46	rep_pSC101_^ts^ Ap^R^ P_BAD_ *gam bet exo*	K.A.Datsenko
pCP20	rep_pSC101_^ts^ Ap^R^ Cm^R^ *cI857* λP_R_ *flp*	K.A.Datsenko

All experiments were performed three times for every bacterial strain, on three different days. After the cells were harvested for each culture condition, a portion of the pellets was lysed for RNA isolation and the remainder cell pellets were washed twice with Cellgro Dulbecco's phosphate-buffered saline (PBS; Mediatech) and finally pelleted in microcentrifuge tubes to an approximate wet weight of 0.1 g/tube. The cells were frozen and stored at −80°C until needed.

### Genetic manipulation of chromosomal genes of *Salmonella* Typhimurium

The primers used for deleting or tagging chromosomal genes of *Salmonella* Typhimurium are listed in Table S5. The procedures of gene deleting and tagging were the same as those described previously [28], [49]. Non-polar in-frame gene deletion was carried out to eliminate the entire coding region except the initiation codon and last 7 amino acids of *smpB* and *hfq*. For HA tagging, pKD13 was modified to encode in-frame double HA tag after the *kan* cassette removal. Two oligonucleotide sequences (2HA-F1 and 2HA-R1) were annealed together as indicated. Using the modified plasmid, pKD13-2HA, as a template, PCR-based chromosomal gene recombination was performed and the sequences encoding 2HA epitopes were inserted in-frame after the last codon (see Table S6 for the scar sequence). All resulting *Salmonella* Typhimurium strains made in this study are listed in Table 2. The deleted mutations were tested in mice for attenuation in virulence and for growth in macrophages. The strains tagged with the HA epitope were used to validate the LC-MS results by Western hybridization using DnaK as a control [50]. In these experiments wells were loaded with equivalent numbers of bacteria.

### Animal experiments

For experimental continuity, 5 six-week-old female BALB/c mice were intraperitoneally (i.p.) infected with 14028s, Δ*smpB*, and Δ*hfq* strains respectively. Bacteria cells were grown in LB overnight, washed with PBS and diluted to 2 colony-forming units (cfu)/µl in PBS. Each mouse was administered 200 cfu i.p. and the dose was enumerated by plating on LB agar media. Mouse mortality was monitored for 21 days and all animal experiments were in accordance with the protocol of Oregon Health & Science University Animal Care and Use Committee.

### Macrophage infection assays

RAW 264.7 macrophage-like cell lines were plated in 24-well plates at 2.5×10^5^ cells/well and incubated overnight at 37°C with 5% CO_2_. The next day, stationary phase bacteria were added at an input multiplicity of infection (m.o.i.) of 50 (resulting in approximately 1 bacteria per cell). Infections were initiated by centrifuging the bacteria onto the cell monolayers at 250×*g* for 5 min and then incubated at 37°C with 5% CO_2_ for 30 min. To remove extracellular bacteria after the infection, the monolayers were washed twice with PBS and supplemented with DMEM plus gentamicin (100 ug/ml final concentration) for 1 h. The presence of gentamicin eliminates all extracellular bacteria. After that time, the cells were washed twice with PBS, overlaid with DMEM plus gentamicin (20 ug/ml final concentration), and incubated at 37°C with 5% CO_2_ for the remainder of the experiment. Macrophage cells were lysed with Triton X-100 for analysis at 1.5 h and 18 h post infection. Colony-forming units (cfu) counts were carried out to evaluate the survival and replication of intracellular bacteria. Briefly, at 1.5 h and 18 h time points, infected cells were washed three times with PBS and lysed by 1% Triton X-100 in PBS. The lysates were collected serially 10-fold diluted, and plated on LB agar plates, and the number of surviving bacteria enumerated following overnight incubation. Intracellular survival assays were performed in triplicate; the value shown is the mean.

### Soluble Protein Preparation

Cell pellets were resuspended in 100 mM NH_4_HCO_3_, pH 8.4 buffer and lysed by using 0.1 mM zirconia/silica beads in a 2.0-ml Cryovial with vigorous vortexing for a total of 3 min with cooling steps. The supernatant and subsequent washes were transferred from the beads into new Cryovials. The beads were repeatedly washed until the supernatant was clear. After protein concentration was determined for the samples, urea and thiourea were added to final concentrations of 7 and 2 M, respectively. Following addition of DTT (5 mM), the samples were incubated at 60°C for 30 min. The samples were then diluted 10-fold with buffer, and CaCl_2_ was added (1 mM) followed by trypsin in a 1∶50 trypsin∶protein ratio. The samples were digested for 3 h at 37°C and subsequently cleaned using a C_18_ solid phase extraction (SPE) column (Supelco). Each 1-ml 100- or 50-mg SPE column was conditioned with MeOH and rinsed with 0.1% TFA in water. Samples were introduced to the columns and then washed with 95∶5 H_2_O∶ACN that contained 0.1% TFA. Excess liquid was removed from the columns under vacuum, and the samples were eluted with 80∶20 ACN∶H_2_O that contained 0.1% TFA and concentrated in a SpeedVac (Thermo-Savant) to a final volume of ∼100 µl. A BCA protein assay was performed to determine peptide concentrations prior to analysis.

### Insoluble Protein Preparation

Cell pellets were treated and lysed as described above for soluble protein preparations. The lysate was centrifuged at 1,300×*g* at 4°C for 2 min, and the supernatant was transferred to polycarbonate ultracentrifuge tubes (Beckman) and centrifuged at 4°C at 356,000×*g* for 10 min. Pellets were resuspended in 50 mM NH_4_HCO_3_, pH 7.8, and ultracentrifuged under the same conditions as used in the previous step. A BCA protein assay was performed on the pellets resuspended in water, and the samples were ultracentrifuged once again (as described above) before discarding the supernatant. Pellets were resuspended in ∼200 µL of a solubilization solution (7 M urea, 2 M thiourea, 1% CHAPS in 50 mM ammonium bicarbonate, pH 7.8), and DTT was added to a final concentration of 9.7 mM. Samples were incubated at 60°C for 30 min, then diluted, and digested in the same manner as described for the global and soluble protein preparation. Samples were cleaned by using an appropriately sized strong cation exchange (SCX) SPE column (Supelco). Each 1-ml 100-mg column was conditioned with MeOH, rinsed in varying sequences and amounts with 10 mM ammonium formate in 25% ACN, pH 3.0; 500 mM ammonium formate in 25% ACN; and Nanopure water. Samples were acidified to a pH of ≤4.0 by adding 20% formic acid followed by centrifugation at 16,000×*g* for 5 min. Samples were then introduced to the columns and washed with 10 mM ammonium formate in 25% ACN, pH 3.0. Excess liquid was removed from the columns under vacuum. The samples were eluted with 80∶15∶5 MeOH∶H_2_O∶NH_4_OH and concentrated to ∼100 µl using a SpeedVac. Final peptide concentrations were determined using a BCA protein assay.

### Capillary Liquid Chromatography (LC)-Mass Spectrometry (MS) Analysis

LC-MS spectra were analyzed using the accurate mass and elution time (AMT) tag approach [51]. Briefly, the theoretical mass and the observed normalized elution time (NET) of each peptide identified by LC-MS/MS is used to construct a reference database of AMT tags, which serve as two-dimensional markers for identifying peptides in subsequent high resolution and high mass accuracy LC-MS analyses. A reference database of AMT tags for *Salmonella* Typhimurium has been generated through exhaustive SCX fractionation and LC-MS/MS analysis as described in our previous publications [52], [53] and the LC-MS/MS data from this experiment, obviating the need for further time consuming SCX LC-MS/MS analyses. This approach to proteomics research is enabled by a number of published and unpublished in-group developed tools, which are available for download at omics.pnl.gov [54], [55], [56], [57], [58]. Prior to the samples being analyzed they were subjected to a blocking and randomization treatment to minimize the effects of systematic biases and ensure the even distribution of known and unknown confounding factors across the entire experimental dataset. Peptides from each of the soluble and insoluble protein preparations were separated by an automated in-house designed reverse-phase capillary HPLC system as described elsewhere [59]. Eluate from the HPLC was directly electrosprayed into an LTQ-Orbitrap mass spectrometer (LTQ-Orbitrap, Thermo Fisher Scientific, San Jose, CA) using electrospray ionization (ESI) with emitters described previously [60] and the ESI interface modified with an electrodynamic ion funnel [61]. Three biological replicates for each sample were analyzed on the ESI interface modified LTQ-Orbitrap mass spectrometer. Relevant information such as the elution time from the capillary LC column, the abundance of the signal (integrated area under the elution profile), and the monoisotopic mass (determined from charge state and the high accuracy *m*/*z* measurement) of each feature observed in the LTQ-Orbitrap was used to match the peptide identifications contained within the AMT tag database. An internally developed algorithm that calculates a probability of match using the mass, normalized elution time, peptide identification values from SEQUEST and their respective distributions was used to match features to peptide identifications [62]. Each peptide used for analysis was observed in at least one LC-MS analysis with a probability of a correct match being 0.9 and matches in the remaining LC-MS analyses were required to be a minimum probability of 0.5, with the additional constraint that at least three peptides with these constraints were required per protein identification. The abundances of these identified peptides are quantified using the chromatographic peak area and were used to infer the protein composition and abundance of the samples. The software program DAnTE [30] was employed to perform this abundance roll-up procedure to convert peptide information into protein information, thereby inferring relative protein abundances. Differences in protein expression between parent and iosgenic mutant was assessed via hypothesis testing, specifically a comprehensive ANOVA scheme included in the software program DAnTE.

### RNA preparation and microarray analysis

Cell pellets from cell harvest above were resuspended in TE buffer pH8.0 containing Lysozyme and processed following the instructions of Qiagen RNeasy midi kit (Qiagen) in combination with DNaseI (Qiagen) treatment. *Salmonella* microarray slide preparation and general microarray analysis followed the procedures described by Porwollik et al. [63]. Total RNA from 14028s, *smpB*, and *hfq* was reverse transcribed with Cy3-linked dUTP and genomic DNA from 14028s was labeled with Cy5-linked dUTP as reference for hybridization. Hybridization mixtures containing same amounts of Cy3-labled probes and Cy5-labeled probes were applied on the *Salmonella* array slide and incubated in a hybridization chamber (Corning) at 42°C overnight. For each strain and condition, three biological repeats were hybridized on separate slides. Slides contained identical triplicate arrays. Slides hybridized with Cy3/Cy5-labled probes were scanned using ScanArray Express software (Packard Bioscience, BioChip Technologies), and the fluorescent signal intensities were quantified using QuantArray (Packard Bioscience, BioChip Technologies). The raw data was processed in WebArray [64], using print-tip Loess normalization within slides, and Scale normalization procedures between slides. The *Salmonella* data discussed in this publication have been deposited in NCBI's Gene Expression Omnibus and are accessible through GEO Series accession number GSE11486.

### Immunoblot analysis

Chromosomal haemaglutanin (HA)-tagged *Salmonella* Typhimurium 14028 wildtype and mutant strains were grown as described above, and the final optical density of each culture was recorded. Cells corresponding to an OD600 of 0.05 were harvested by centrifugation at 10000 *g* in a microfuge at 4°C, washed twice with an equal volume of ice-cold DPBS, resuspended in 50 µL of Laemmli 1× sample buffer, and boiled for 5 min. A 1/10th volume of the total cell lysate was loaded into the wells of 15% Tris-Cl sodium dodecyl sulfate-polyacrylamide gel electrophoresis (SDS-PAGE) gels (BIO-RAD). After the proteins were separated on the gel, the proteins were electrophoretically transferred to polyvinylidene difluoride (PVDF) membranes (Millipore). The membranes were blocked in Tris-buffered saline (TBS) plus 5% powdered nonfat dry milk for 1 h, probed with an anti-HA monoclonal antibody (Covance, 1∶1,000 dilution in block solution) and an anti-DnaK monoclonal antibody (Stressgen, 1∶4,000 dilution in block solution) for 1 h, washed 3 times for 5 min with TBS, probed with a goat-antimouse 2° antibody (Sigma A4416, 1∶5000 dilution in block solution) for 30 min, and finally washed 3 times for 5 min with TBS. The immune complexes were detected via chemiluminescence using Perkin-Elmer's “Western Lightning” reagents and then exposed to XAR Biofilm (Kodak).

### Quantitative PCR analysis

Wild-type strain and mutant strain cells were grown as described above and harvested for RNA extraction. Total RNA was isolated using RNAprotect™ (Qiagen), RNeasy kit (Qiagen), and DNase set (Qiagen) according to the manufacturer's recommendations. cDNA was synthesized using iScript cDNA systhesis kit (Bio Rad) and subjected to real-time PCR (ABI 7700, Applied Biosystems) with SYBR green PCR master mix (Applied Biosystems) and primer sets. Primers used in RT-PCR are listed in Table S5. The expression ratio of each gene is the average from three independent RNA samples and was normalized to the level of *gyrB*. *gyrB* is assumed to be a steadily transcribed housekeeping gene.

## Supporting Information

Table S1 List of gene products (proteins) regulated by Hfq and gene products (proteins) regulated by SmpB under the growth conditions examined.
(1.17 MB XLS)

Table S2 List of proteins that overlap between Hfq-regulated proteins and Hfq-associated mRNAs.
(0.07 MB XLS)

Table S3 Validation of proteomics dataset by cross-comparison with the literature.
(0.05 MB XLS)

Table S4 List of gene transcripts (RNA) regulated by Hfq and gene transcripts (RNA) regulated by SmpB under the growth conditions examined.
(0.23 MB XLS)

Table S5 List of primers used in this study.
(0.05 MB DOC)

Table S6 Scar sequences.
(0.03 MB DOC)

Figure S1 Growth curves of strains in LB and minimal acidic media: Growth phenotypes of wild-type (open corcle), Δ*smpB* (closed square), and Δ*hfq* (closed triangle) were compared in LB (A) and minimal acidic (B) media. For the growth in minimal acidic media, cells were pre-cultured in MgM (pH5.0) overnight and diluted 1∶100 into fresh MgM (pH5.0) media.
(0.77 MB TIF)

Figure S2 Immunoblot analysis of the protein levels of selected proteins from varying *Salmonella* wildtype and mutant strain cultures: *Salmonella* wildtype and Δ*hfq* mutant strains harboring HA-tag at the HtrA, STM1513 and OsmY genes (Panel A) and *Salmonella* wildtype and Δ*smpB* mutant strains harboring HA-tag at the FliC and YciF genes (Panel B) were grown under the indicated conditions as described. Same amount of cell lysates was loaded in each lane and probed by Western blot analysis for the indicated proteins and a control protein DnaK. AMM = Acidic minimal media. The level of YciF-encoded protein expressed in each strain was normalized to the DnaK level. Intracellular DnaK level in each lane was set to 100% (Panel C).
(2.35 MB TIF)
