# Supplementary material for: Global Systems-Level Analysis of Hfq and SmpB Deletion Mutants in Salmonella: Implications for Virulence and Global Protein Translation
Source: PLoS One. 2009 Mar 11;4(3):e4809. doi: 10.1371/journal.pone.0004809 (PMC2652828; doi:10.1371/journal.pone.0004809)
Supplement: Table S5 — List of primers used in this study. (0.05 MB DOC) [file pone.0004809.s005.doc]

**Table S5.** List of primers used in this study.

| Primers | Sequences (5’ - end to 3’ - end) | Purpose |
| --- | --- | --- |
| STM2688-RF1 | TTTTCGATATCAGATTACCTATGAATTCACGACACTTATGATTCCGGGGATCCGTCGACC | Δ *smpB* |
| STM2688-RR1 | TCACAAAAAATGTCTATTATCGGCCTGCATTTTTCATAATGTGTAGGCTGGAGCTGCTCC | Δ *smpB* |
| STM4361-RF1 | AGGTTCAAAGTACAAATAAGCATATAAGGAAAAGAGAATGATTCCGGGGATCCGTCGACC | Δ *hfq* |
| STM4361-RR1 | TAAACAGCGCGTGAACTTATTCAGTCTCTTCGCTGTCCTGGTGTAGGCTGGAGCTGCTCC | Δ *hfq* |
| 2HA-F1 | CATATCCATATGACGTCCCAGACTACGCCAGCGGATACCCATACGATGTTCCAGATTACGCTC | pKD13-2HA |
| 2HA-R1 | CTAGGAGCGTAATCTGGAACATCGTATGGGTATCCGCTGGCGTAGTCTGGGACGTCATATGGATATGAGCT | pKD13-2HA |
| pduE-HARF1 | AGCTTTCGTTCGTGAAGCGGCAGGGCTGTACGTTGAGCGTATTCCGGGGATCCGTCGACC | PduE-2HA |
| pduE-HARR1 | ATCGCATACGAAATCCTTAATCGTCGCCTTTGAGTTTTTTGTGTAGGCTGGAGCTGCTCC | PduE-2HA |
| pduA-HARF1 | ACACGTCATCCCACGCCCTCACACCGATGTAGAAAAAATCATTCCGGGGATCCGTCGACC | PduA-2HA |
| pduA-HARR1 | ACCAGCTCATTGCTGCTCATTGGCTAATTCCCTTCGGTAAGTGTAGGCTGGAGCTGCTCC | PduA-2HA |
| htrA-HARF1 | GCCGTCGGTTCTGGCGCTGAATATTCAGCGTGGTGATAGTATTCCGGGGATCCGTCGACC | HtrA-2HA |
| htrA-HARR1 | AGGGGGACAAAGGTGATTACTGCATCAGCAAATAAATAGAGTGTAGGCTGGAGCTGCTCC | HtrA-2HA |
| STM1513-HARF1 | ATCTGAAGCGGGTAAAAAAGGTGGCCAAAATAGCCACAGTATTCCGGGGATCCGTCGACC | STM1513-2HA |
| STM1513-HARR1 | AATATCAGCAGTAAAATTAATTGCCGGATTTCCGTCCACCGTGTAGGCTGGAGCTGCTCC | STM1513-2HA |
| osmY-HARF1 | TGAAAGCATCGCGAAAGCCGTTGATGGCGTAAAAAGTGTTATTCCGGGGATCCGTCGACC | OsmY-2HA |
| osmY-HARR1 | GACGAATTACGACGAATTACTGAACTTTCAGATCGTTTTTGTGTAGGCTGGAGCTGCTCC | OsmY-2HA |
| fliC-HARF1 | CGGTACCTCCGTTCTGGCGCAGGCGAACCAGGTTCCGCAAATTCCGGGGATCCGTCGACC | FliC-2HA |
| fliC-HARR1 | TGAATCAATCGCCGGATTAACGCAGTAAAGAGAGGACGTTGTGTAGGCTGGAGCTGCTCC | FliC-2HA |
| yciF-HARF1 | TTTAAAACTTACCGATTTAGCAGTCAGCAATGTTAATAAAATTCCGGGGATCCGTCGACC | YciF-2HA |
| yciF-HARR1 | CTGATAATTAAAAATATTATTTCGATTTGCGTTCAGCACTGTGTAGGCTGGAGCTGCTCC | YciF-2HA |
| pduE-F1 | GGACGACTTTACGCTGGAAAAC | qRT-PCR |
| pduE-R1 | TTGGCGATAGAGGCCTGTAAG | qRT-PCR |
| pduA-F1 | GGAATGGTAGAAACCAAAGGCTTA | qRT-PCR |
| pduA-R1 | GCCAATCTTTTCATAGCCCACTA | qRT-PCR |
