# Supplementary material for: Global Systems-Level Analysis of Hfq and SmpB Deletion Mutants in Salmonella: Implications for Virulence and Global Protein Translation
Source: PLoS One. 2009 Mar 11;4(3):e4809. doi: 10.1371/journal.pone.0004809 (PMC2652828; doi:10.1371/journal.pone.0004809)
Supplement: Table S6 — Scar sequences. (0.03 MB DOC) [file pone.0004809.s006.doc]

**Table S6.** Scar sequences.

| ATTCCGGGGATCCGTCGACCTGCAGTTCGAAGTTCCTATTCTCTAGAAAGTA  Priming site 4 HA-tag |
| --- |
| TAGGAACTTCGAGCTCATATCCATATGACGTCCCAGACTACGCCAGCGGAT  SacI |
| ACCCATACGATGTTCCAGATTACGCTCCTAGGAGCAGCTCCAGCCTACAC  HA-tag AvrII Priming site 1 |
