# Supplementary figures and images for: Global Systems-Level Analysis of Hfq and SmpB Deletion Mutants in Salmonella: Implications for Virulence and Global Protein Translation
Source: PLoS One. 2009 Mar 11;4(3):e4809. doi: 10.1371/journal.pone.0004809 (PMC2652828; doi:10.1371/journal.pone.0004809)

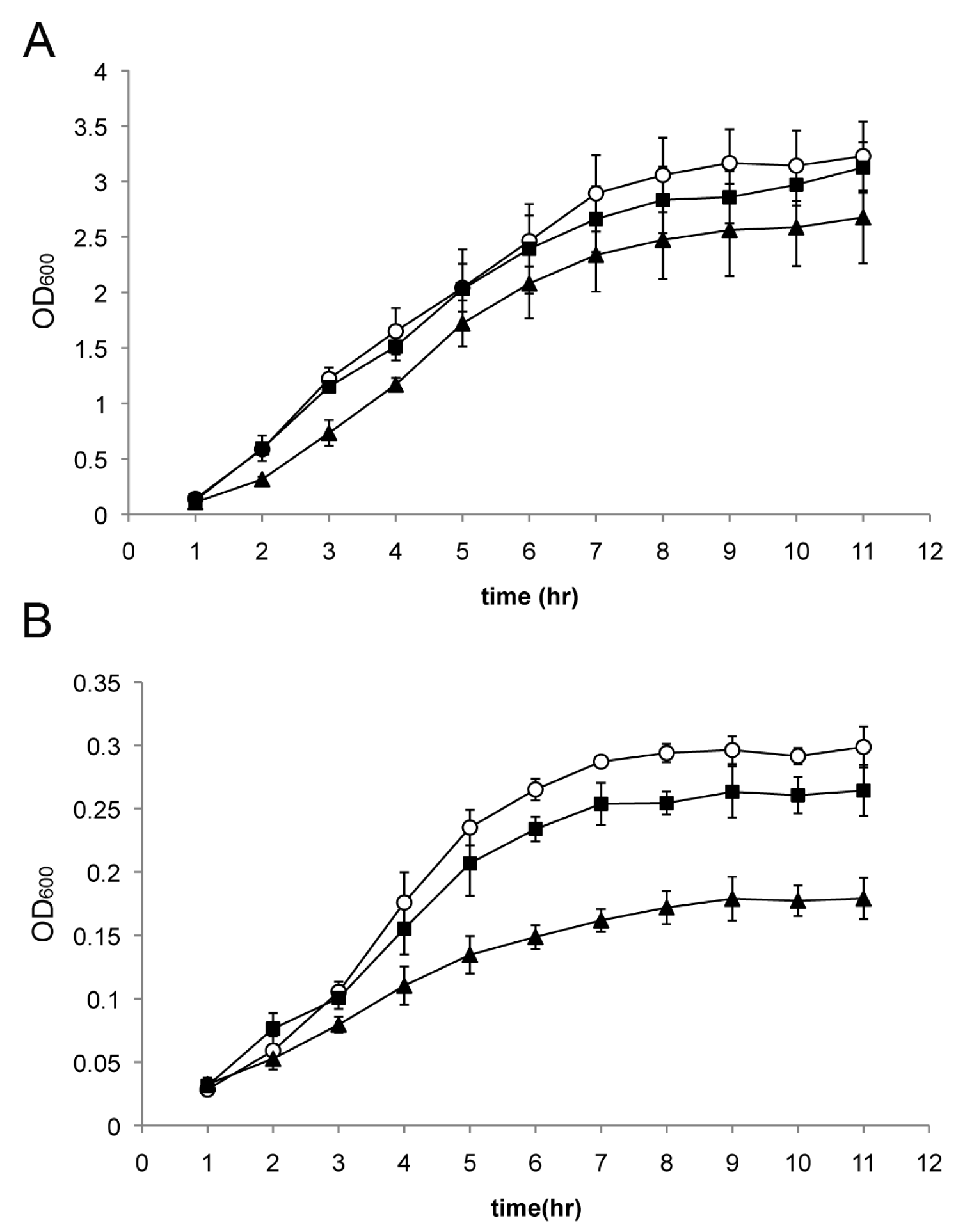

Supplement: Figure S1 — Growth curves of strains in LB and minimal acidic media: Growth phenotypes of wild-type (open corcle), ΔsmpB (closed square), and Δhfq (closed triangle) were compared in LB (A) and minimal acidic (B) media. For the growth in minimal acidic media, cells were pre-cultured in MgM (pH5.0) overnight and diluted 1∶100 into fresh MgM (pH5.0) media. (0.77 MB TIF) [file pone.0004809.s007.tif]

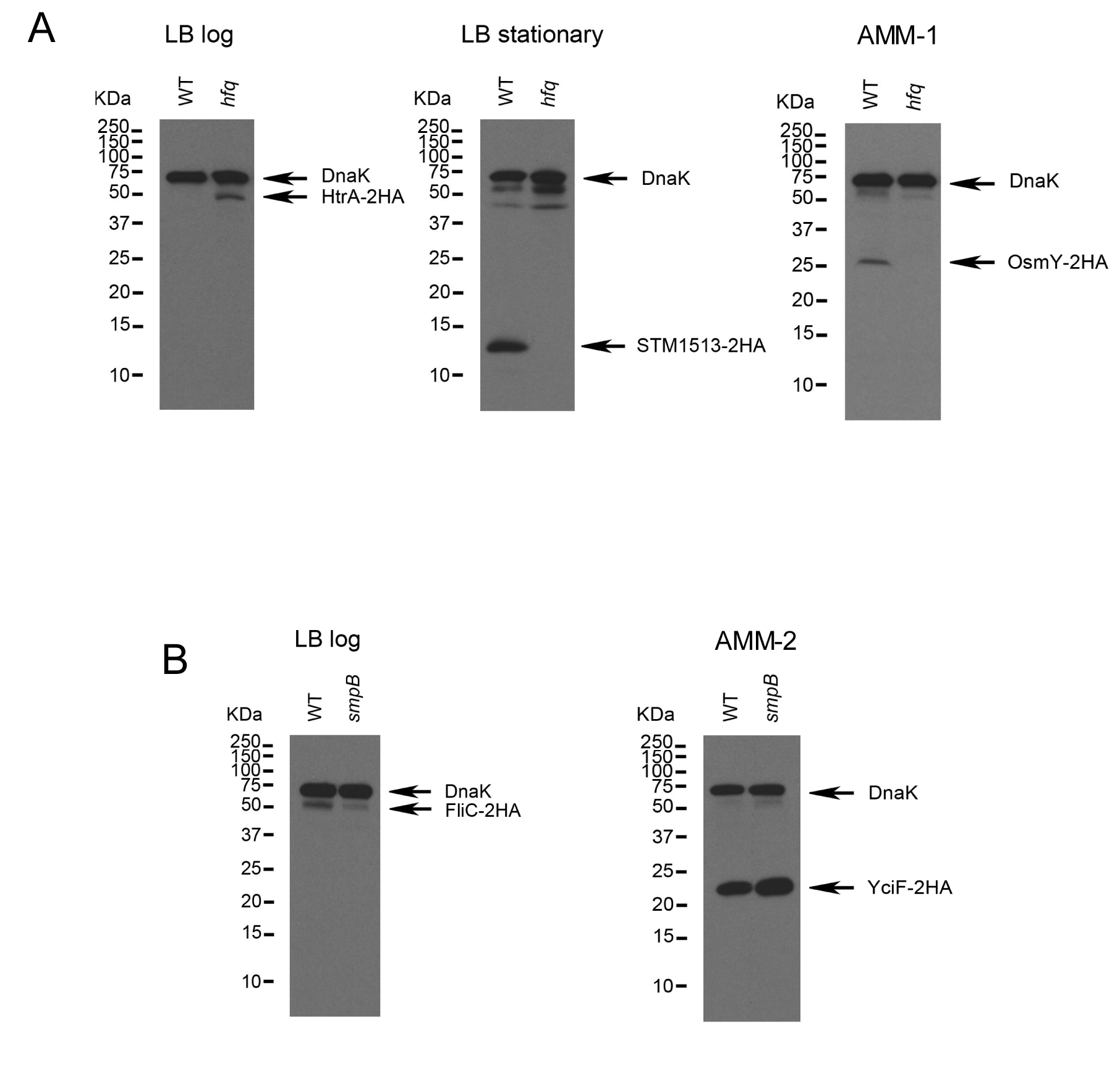

Supplement: Figure S2 — Immunoblot analysis of the protein levels of selected proteins from varying Salmonella wildtype and mutant strain cultures: Salmonella wildtype and Δhfq mutant strains harboring HA-tag at the HtrA, STM1513 and OsmY genes (Panel A) and Salmonella wildtype and ΔsmpB mutant strains harboring HA-tag at the FliC and YciF genes (Panel B) were grown under the indicated conditions as described. Same amount of cell lysates was loaded in each lane and probed by Western blot analysis for the indicated proteins and a control protein DnaK. AMM = Acidic minimal media. The level of YciF-encoded protein expressed in each strain was normalized to the DnaK level. Intracellular DnaK level in each lane was set to 100% (Panel C). (2.35 MB TIF) [file pone.0004809.s008.tif]
